# Supplementary material for: Zoonotic Implications of the Co-Circulation of Clade 2.3.4.4b and 2.3.2.1a H5N1 Avian Influenza Viruses in Nepal in 2023
Source: Viruses. 2025 Nov 6;17(11):1481. doi: 10.3390/v17111481 (PMC12656878; doi:10.3390/v17111481)
Supplement: Supplementary file 1 [file viruses-17-01481-s001.zip › viruses-3933388-supplementary.pdf]

**A**

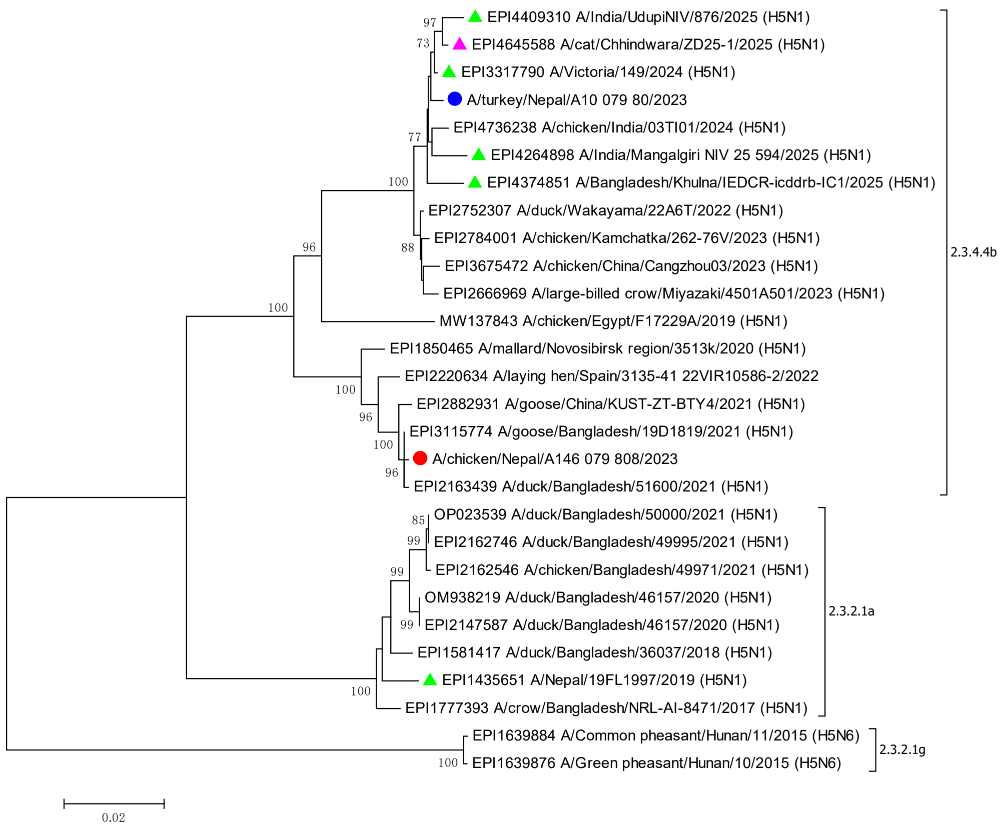
EPI4409310 A/India/UdupiNIV/876/2025 (H5N1)
EPI4645588 A/cat/Chhindwara/ZD25-1/2025 (H5N1)
EPI3317790 A/Victoria/149/2024 (H5N1)
A/turkey/Nepal/A10 079 80/2023
EPI4736238 A/chicken/India/03TI01/2024 (H5N1)
EPI4264898 A/India/Mangalgiri NIV 25 594/2025 (H5N1)
EPI4374851 A/Bangladesh/Khulna/IEDCR-icddrb-IC1/2025 (H5N1)
EPI2752307 A/duck/Wakayama/22A6T/2022 (H5N1)
EPI2784001 A/chicken/Kamchatka/262-76V/2023 (H5N1)
EPI3675472 A/chicken/China/Cangzhou03/2023 (H5N1)
EPI2666969 A/large-billed crow/Miyazaki/4501A501/2023 (H5N1)
MW137843 A/chicken/Egypt/F17229A/2019 (H5N1)
EPI1850465 A/mallard/Novosibirsk region/3513k/2020 (H5N1)
EPI2220634 A/laying hen/Spain/3135-41 22VIR10586-2/2022
EPI2882931 A/goose/China/KUST-ZT-BTY4/2021 (H5N1)
EPI3115774 A/goose/Bangladesh/19D1819/2021 (H5N1)
A/chicken/Nepal/A146 079 808/2023
EPI2163439 A/duck/Bangladesh/51600/2021 (H5N1)
OP023539 A/duck/Bangladesh/50000/2021 (H5N1)
EPI2162746 A/duck/Bangladesh/49995/2021 (H5N1)
EPI2162546 A/chicken/Bangladesh/49971/2021 (H5N1)
OM938219 A/duck/Bangladesh/46157/2020 (H5N1)
EPI2147587 A/duck/Bangladesh/46157/2020 (H5N1)
EPI1581417 A/duck/Bangladesh/36037/2018 (H5N1)
EPI1435651 A/Nepal/19FL1997/2019 (H5N1)
EPI1777393 A/crow/Bangladesh/NRL-AI-8471/2017 (H5N1)
EPI1639884 A/Common pheasant/Hunan/11/2015 (H5N6)
EPI1639876 A/Green pheasant/Hunan/10/2015 (H5N6)
2.3.4.4b
2.3.2.1a
2.3.2.1g
97
73
77
100
88
96
100
100
96
100
96
85
99
99
99
100
100
0.02

**B**

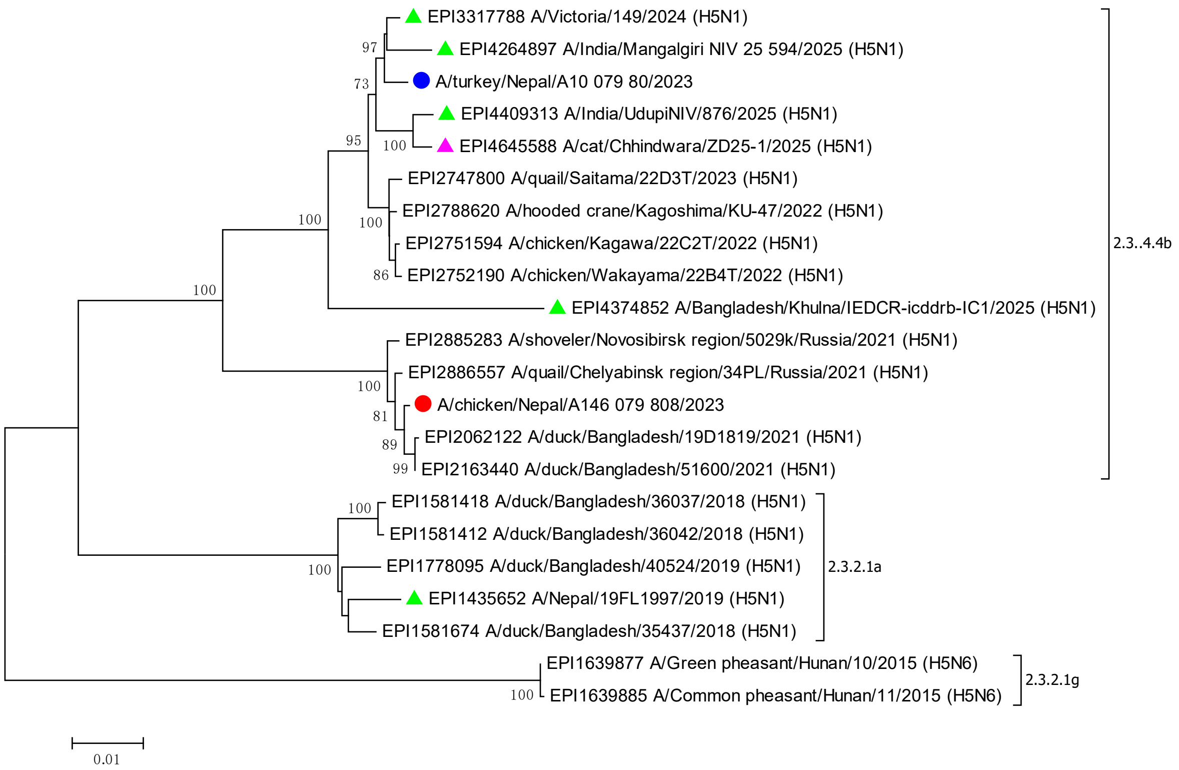
EPI3317788 A/Victoria/149/2024 (H5N1)
EPI4264897 A/India/Mangalgiri NIV 25 594/2025 (H5N1)
A/turkey/Nepal/A10 079 80/2023
EPI4409313 A/India/UdupiNIV/876/2025 (H5N1)
EPI4645588 A/cat/Chhindwara/ZD25-1/2025 (H5N1)
EPI2747800 A/quail/Saitama/22D3T/2023 (H5N1)
EPI2788620 A/hooded crane/Kagoshima/KU-47/2022 (H5N1)
EPI2751594 A/chicken/Kagawa/22C2T/2022 (H5N1)
EPI2752190 A/chicken/Wakayama/22B4T/2022 (H5N1)
EPI4374852 A/Bangladesh/Khulna/IEDCR-icddrb-IC1/2025 (H5N1)
EPI2885283 A/shoveler/Novosibirsk region/5029k/Russia/2021 (H5N1)
EPI2886557 A/quail/Chelyabinsk region/34PL/Russia/2021 (H5N1)
A/chicken/Nepal/A146 079 808/2023
EPI2062122 A/duck/Bangladesh/19D1819/2021 (H5N1)
EPI2163440 A/duck/Bangladesh/51600/2021 (H5N1)
EPI1581418 A/duck/Bangladesh/36037/2018 (H5N1)
EPI1581412 A/duck/Bangladesh/36042/2018 (H5N1)
EPI1778095 A/duck/Bangladesh/40524/2019 (H5N1)
EPI1435652 A/Nepal/19FL1997/2019 (H5N1)
EPI1581674 A/duck/Bangladesh/35437/2018 (H5N1)
EPI1639877 A/Green pheasant/Hunan/10/2015 (H5N6)
EPI1639885 A/Common pheasant/Hunan/11/2015 (H5N6)
2.3..4.4b
2.3.2.1a
2.3.2.1g
97
73
95
100
100
100
86
100
100
81
89
99
100
100
100
0.01

**C**

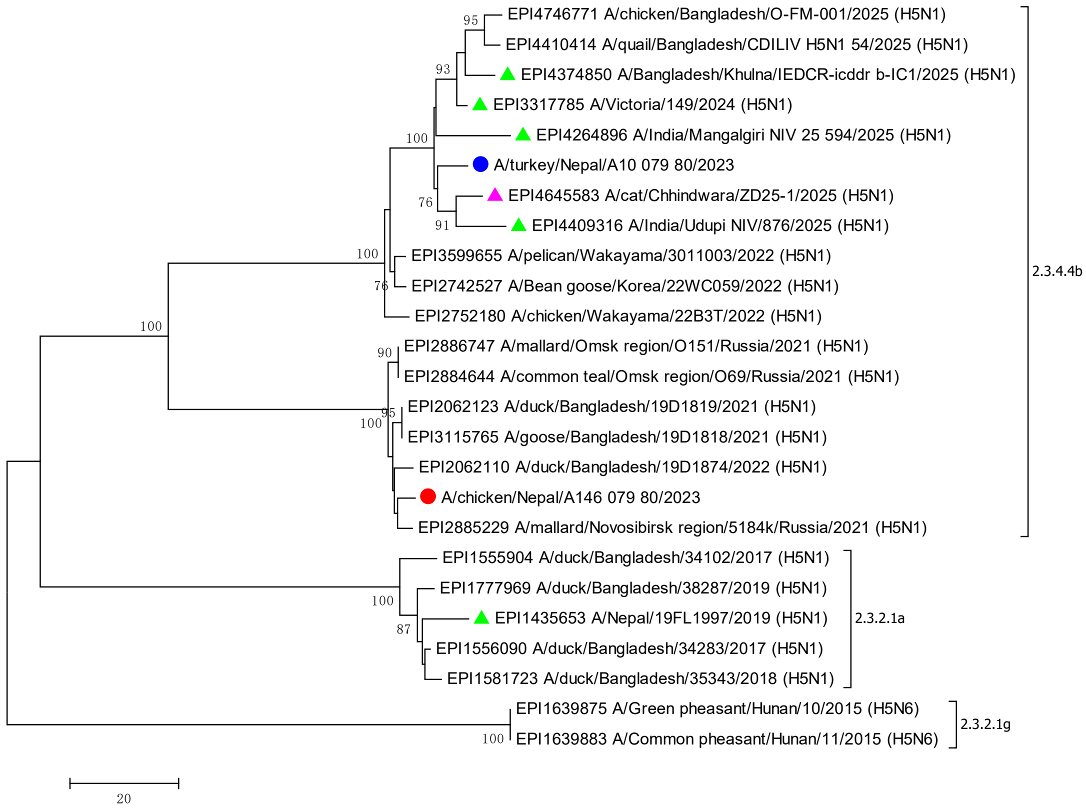
EPI4746771 A/chicken/Bangladesh/O-FM-001/2025 (H5N1)
EPI4410414 A/quail/Bangladesh/CDILIV H5N1 54/2025 (H5N1)
EPI4374850 A/Bangladesh/Khulna/IEDCR-icddr b-IC1/2025 (H5N1)
EPI3317785 A/Victoria/149/2024 (H5N1)
EPI4264896 A/India/Mangalgiri NIV 25 594/2025 (H5N1)
A/turkey/Nepal/A10 079 80/2023
EPI4645583 A/cat/Chhindwara/ZD25-1/2025 (H5N1)
EPI4409316 A/India/Udupi NIV/876/2025 (H5N1)
EPI3599655 A/pelican/Wakayama/3011003/2022 (H5N1)
EPI2742527 A/Bean goose/Korea/22WC059/2022 (H5N1)
EPI2752180 A/chicken/Wakayama/22B3T/2022 (H5N1)
EPI2886747 A/mallard/Omsk region/O151/Russia/2021 (H5N1)
EPI2884644 A/common teal/Omsk region/O69/Russia/2021 (H5N1)
EPI2062123 A/duck/Bangladesh/19D1819/2021 (H5N1)
EPI3115765 A/goose/Bangladesh/19D1818/2021 (H5N1)
EPI2062110 A/duck/Bangladesh/19D1874/2022 (H5N1)
A/chicken/Nepal/A146 079 80/2023
EPI2885229 A/mallard/Novosibirsk region/5184k/Russia/2021 (H5N1)
EPI1555904 A/duck/Bangladesh/34102/2017 (H5N1)
EPI1777969 A/duck/Bangladesh/38287/2019 (H5N1)
EPI1435653 A/Nepal/19FL1997/2019 (H5N1)
EPI1556090 A/duck/Bangladesh/34283/2017 (H5N1)
EPI1581723 A/duck/Bangladesh/35343/2018 (H5N1)
EPI1639875 A/Green pheasant/Hunan/10/2015 (H5N6)
EPI1639883 A/Common pheasant/Hunan/11/2015 (H5N6)
2.3.4.4b
2.3.2.1a
2.3.2.1g
95
93
100
76
91
100
76
100
90
95
100
100
87
100
20

**D**

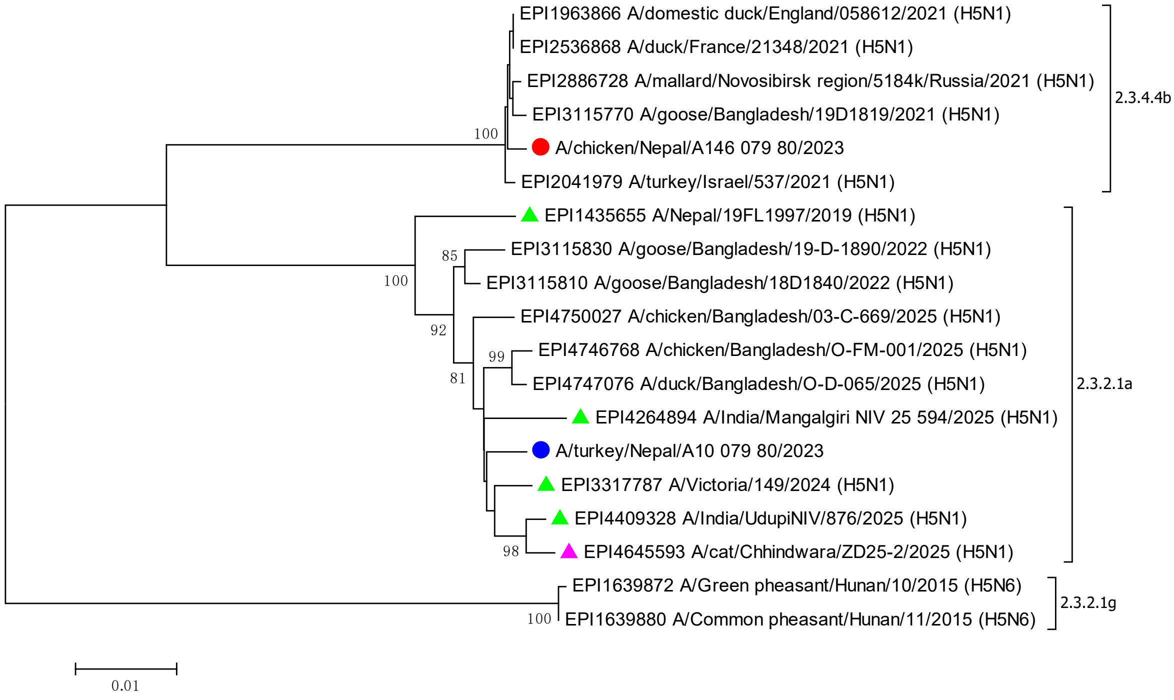
EPI1963866 A/domestic duck/England/058612/2021 (H5N1)
EPI2536868 A/duck/France/21348/2021 (H5N1)
EPI2886728 A/mallard/Novosibirsk region/5184k/Russia/2021 (H5N1)
EPI3115770 A/goose/Bangladesh/19D1819/2021 (H5N1)
A/chicken/Nepal/A146 079 80/2023
EPI2041979 A/turkey/Israel/537/2021 (H5N1)
EPI1435655 A/Nepal/19FL1997/2019 (H5N1)
EPI3115830 A/goose/Bangladesh/19-D-1890/2022 (H5N1)
EPI3115810 A/goose/Bangladesh/18D1840/2022 (H5N1)
EPI4750027 A/chicken/Bangladesh/03-C-669/2025 (H5N1)
EPI4746768 A/chicken/Bangladesh/O-FM-001/2025 (H5N1)
EPI4747076 A/duck/Bangladesh/O-D-065/2025 (H5N1)
EPI4264894 A/India/Mangalgiri NIV 25 594/2025 (H5N1)
A/turkey/Nepal/A10 079 80/2023
EPI3317787 A/Victoria/149/2024 (H5N1)
EPI4409328 A/India/UdupiNIV/876/2025 (H5N1)
EPI4645593 A/cat/Chhindwara/ZD25-2/2025 (H5N1)
EPI1639872 A/Green pheasant/Hunan/10/2015 (H5N6)
EPI1639880 A/Common pheasant/Hunan/11/2015 (H5N6)
2.3.4.4b
2.3.2.1a
2.3.2.1g
100
85
100
92
99
81
98
100
0.01

E

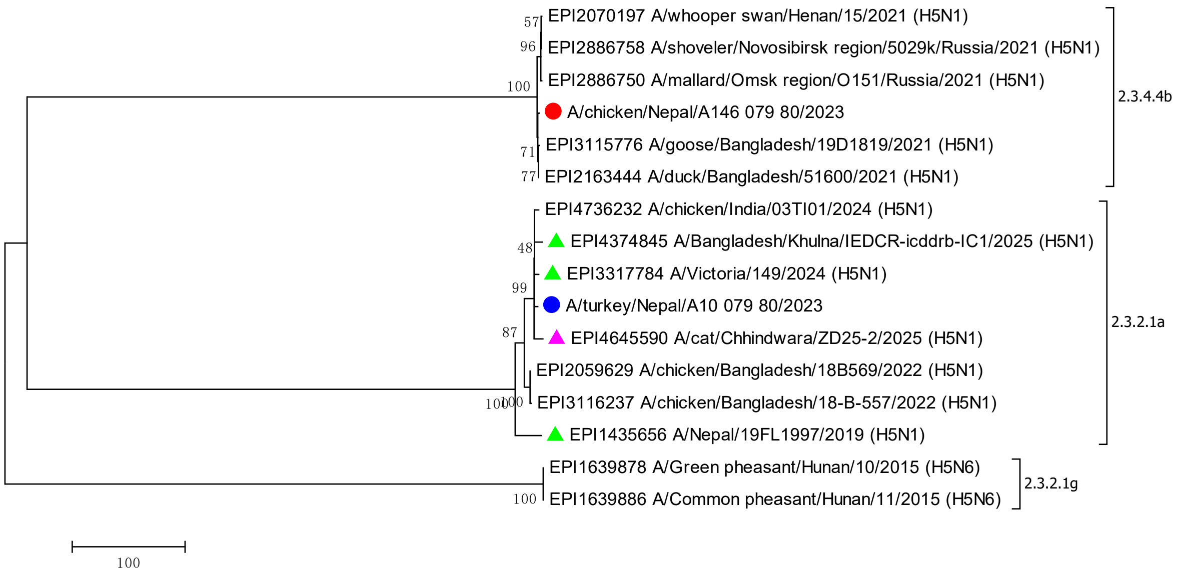
EPI2070197 A/whooper swan/Henan/15/2021 (H5N1)
EPI2886758 A/shoveler/Novosibirsk region/5029k/Russia/2021 (H5N1)
EPI2886750 A/mallard/Omsk region/O151/Russia/2021 (H5N1)
A/chicken/Nepal/A146 079 80/2023
EPI3115776 A/goose/Bangladesh/19D1819/2021 (H5N1)
EPI2163444 A/duck/Bangladesh/51600/2021 (H5N1)
2.3.4.4b
EPI4736232 A/chicken/India/03TI01/2024 (H5N1)
EPI4374845 A/Bangladesh/Khulna/IEDCR-icddrb-IC1/2025 (H5N1)
EPI3317784 A/Victoria/149/2024 (H5N1)
A/turkey/Nepal/A10 079 80/2023
EPI4645590 A/cat/Chhindwara/ZD25-2/2025 (H5N1)
EPI2059629 A/chicken/Bangladesh/18B569/2022 (H5N1)
EPI3116237 A/chicken/Bangladesh/18-B-557/2022 (H5N1)
EPI1435656 A/Nepal/19FL1997/2019 (H5N1)
2.3.2.1a
EPI1639878 A/Green pheasant/Hunan/10/2015 (H5N6)
EPI1639886 A/Common pheasant/Hunan/11/2015 (H5N6)
2.3.2.1g
100

F

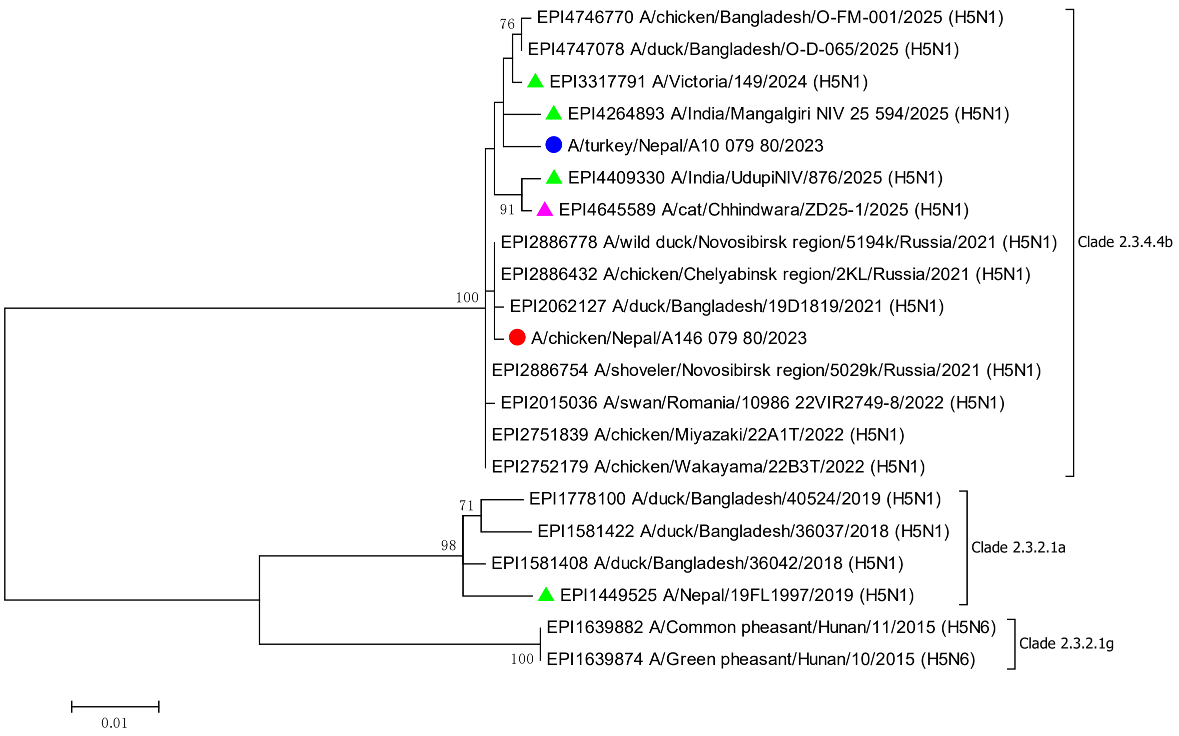
EPI4746770 A/chicken/Bangladesh/O-FM-001/2025 (H5N1)
EPI4747078 A/duck/Bangladesh/O-D-065/2025 (H5N1)
EPI3317791 A/Victoria/149/2024 (H5N1)
EPI4264893 A/India/Mangalgiri NIV 25 594/2025 (H5N1)
A/turkey/Nepal/A10 079 80/2023
EPI4409330 A/India/UdupiNIV/876/2025 (H5N1)
EPI4645589 A/cat/Chhindwara/ZD25-1/2025 (H5N1)
EPI2886778 A/wild duck/Novosibirsk region/5194k/Russia/2021 (H5N1)
EPI2886432 A/chicken/Chelyabinsk region/2KL/Russia/2021 (H5N1)
EPI2062127 A/duck/Bangladesh/19D1819/2021 (H5N1)
A/chicken/Nepal/A146 079 80/2023
EPI2886754 A/shoveler/Novosibirsk region/5029k/Russia/2021 (H5N1)
EPI2015036 A/swan/Romania/10986 22VIR2749-8/2022 (H5N1)
EPI2751839 A/chicken/Miyazaki/22A1T/2022 (H5N1)
EPI2752179 A/chicken/Wakayama/22B3T/2022 (H5N1)
Clade 2.3.4.4b
EPI1778100 A/duck/Bangladesh/40524/2019 (H5N1)
EPI1581422 A/duck/Bangladesh/36037/2018 (H5N1)
EPI1581408 A/duck/Bangladesh/36042/2018 (H5N1)
EPI1449525 A/Nepal/19FL1997/2019 (H5N1)
Clade 2.3.2.1a
EPI1639882 A/Common pheasant/Hunan/11/2015 (H5N6)
EPI1639874 A/Green pheasant/Hunan/10/2015 (H5N6)
Clade 2.3.2.1g
0.01

# G

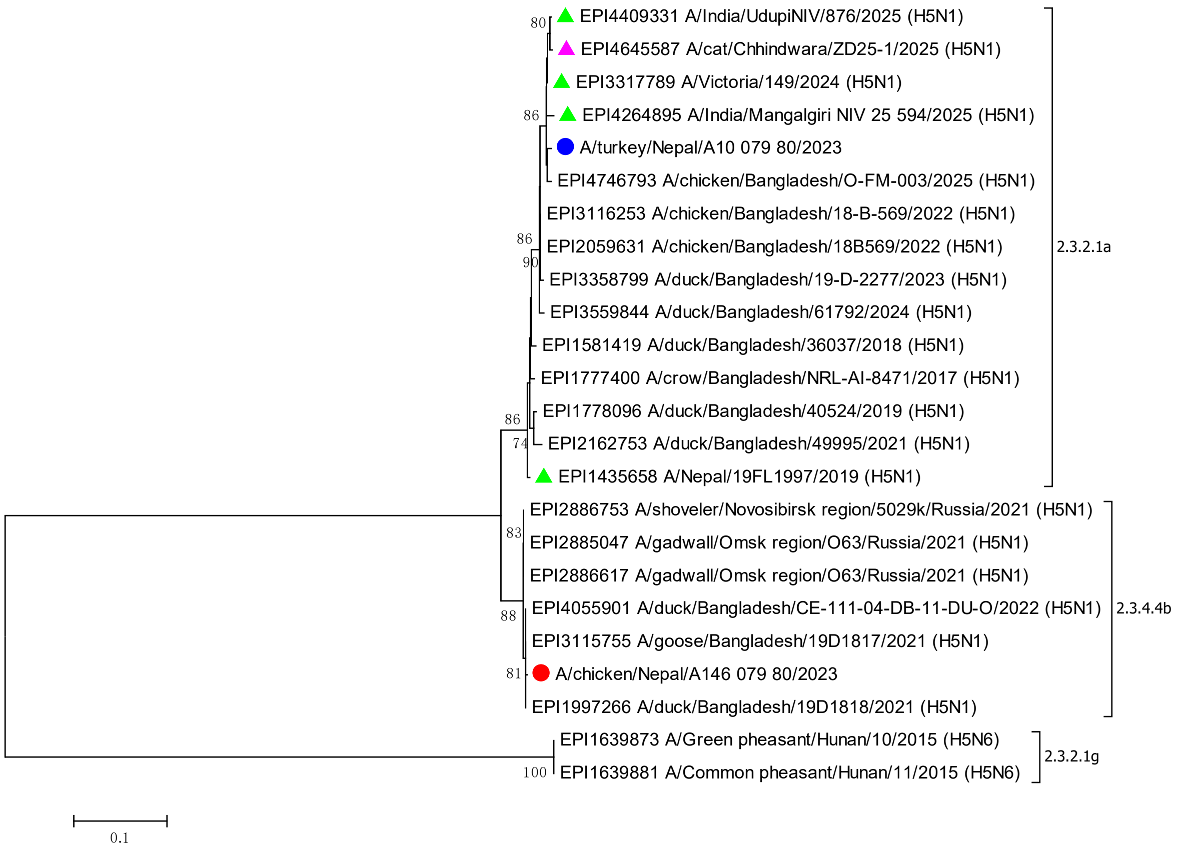


**Figure S1** Maximum-likelihood phylogenetic tree of a (**A**) PB2 (**B**) PB1, (**C**) PA, (**D**) NP, (**E**) NA, (**F**) M and (**G**) NS segments. The two samples from this study are highlighted with a red and blue dot. Human and feline samples are shown with green and pink triangles respectively. Clades are identified and bootstrap values >70 are shown.

Supplementary File S1: Avian influenza virus subtype N1 protocol



## Avian Influenza Virus Subtype N1 Quick Reference Sheet

| General Information | |
|---|---|
| **Taxonomy** | Genus *influenza A*, family *Orthomyxoviridae* |
| **Genome** | Segmented negative sense single stranded RNA |
| **Host species** | Avian |
| **Reference** | Wang et al unpublished (In-house AAHL AI N1 assay) |
| **CAUTION!!** | **Zoonosis. Potential Human pathogen** |
| **Suitable sample** | Tracheal and cloacal swabs or faeces, tissue homogenates, pools of organs, allantoic fluid or others as deemed appropriate. |

| Real-Time TaqMan PCR Assays | |
|---|---|
| **Gene target** | NA gene of avian influenza virus. This assay also detects pandemic H1N1 (2009) influenza A virus. |
| **Recommendations for use** | Is not intended as a primary detection assay. Can be used as a follow-up assay after confirmation as positive in Matrix and H5 PCR assay systems.<br>Positive, uncharacteristic and late cycle curve amplification needs to be followed up with nucleotide sequence. |
| **AI N1 Assay** | **AI N1 PP Mix**<br>Assay is provided as 80uL liquid. **Add 720uL RNase free water, Use 1.5uL per reaction** |

| Primer and probe details | Name | Sequence | FINAL Concentration |
|---|---|---|---|
| | AI N1 1316Fm Fwd: | 5'- GYGGGAGCAGCMTWTCYTT-3' | 800nM |
| | AI N1 1379R Rev: | 5'-CCGTCTGGCCAAGACCAA-3' | 800nM |
| | AI N1 1336Pm Probe: | 5'- FAM-TGTGGTGTAAAYAGTGAMAC-MGB-3' | 200nM |

| | | |
|---|---|---|
| **Thermal cycler program** | 1 x 45°C 10 min, 95°C 10 min<br>45 x 95°C 15 sec, 60°C 45 sec | **These are standard AgPath amplification conditions. If other PCR reagents are used these conditions will need to be modified according to the manufacturers guidelines.** |
| **Positive controls** | H5N1 strong and weak positive extraction controls | |

| Result Analysis and Interpretation | |
|---|---|
| **Threshold setting** | 0.1 (ABI 7500) |
| **Interpretation** | Positive: Ct less than 40; Having a characteristic amplification curve<br>Negative: Ct greater than 45; Ct undetectable; No characteristic amplification curve<br>Indeterminate: Ct between 40 and 45 |

## Influenza A N1 RT-PCR Master Mix Sheet (AI N1)

Samples:______________________________ Lab Code:______________

Extraction Operator: ______________________ Extraction Date: ______________

Extraction Kit used:______________________ Extraction Kit Batch:______________

PCR Operator: ______________________ PCR date:______________________

| REACTION COMPONENT | VOLUME PER REACTION (μL) | VOLUME FOR ....... REACTIONS (μL) |
|---|---|---|
| Nuclease Free water | 0.4 | |
| **2X AgPath RT-PCR Buffer** (Ambion P/N AM1005)<br>Lot #: ............................................ | 7.5 | |
| 25X RT PCR Enzyme Mix | 0.6 | |
| **AI N1 PP Mix**<br>Batch #: ............................................ | 1.5 | |
| **Total volume** | **10.0** | |
| Template RNA | 5.0 | |

| **AgPath Cycling Parameters** | Standard AgPath amplification conditions. Note cycling parameters may differ when using different kits | Reporter Dye: FAM<br>Quencher: MGB<br>Set Quencher as **MGB** |
|---|---|---|
| 1X 45°C 10 min, 95°C 10 min<br>45X 95°C 15 sec, 60°C 45 sec | | |

| Quality Control | | | | |
|---|---|---|---|---|
| Control Description: Strong/Weak/NTC/ Extraction Pos or Neg | Batch # | Expected Result (e.g. Ct =29-32, Negative) | Actual result | QC pass/fail |
| | | | | |
| | | | | |
| | | | | |
| | | | | |

**Table S1** Mutations identified in the genomic segments of A/chicken/Nepal/A146_079_808/2023 (**A**), A/turkey/Nepal/A10_079_80/2023 (**B**), and A/Victoria/149/2024 (**C**) according to the FluServer tool available in GISAID. Mutations discussed in the main text are highlighted in red while differences between A/turkey/Nepal/A10_079_80/2023 and A/Victoria/149/2024 are highlighted in yellow.

| Virus | Segment | List of mutations |
|---|---|---|
| **A** | PB2 | N102T, V292I, V356I, M444V |
| **B** | PB2 | I67V, N82S, K116R, V139I, V255L, I292V, V338D, R355G, K389R, L464M, M676A, A717T |
| **C** | PB2 | I67V, N82S, K116R, V139I, V255L, I292V, V338D, K389R, L464M, M676A, A717T |
| | | |
| **A** | PB1 | N16D, R430K, D619G, A652T, S694I, G733R |
| **B** | PB1 | E75G, M317T, Y324F, E581G, M655I, L695I |
| **C** | PB1 | E75G, E97G, K121R, E581G, M655I, L695I |
| | | |
| **A** | PB1-F2 | NONE |
| **B** | PB1-F2 | NONE |
| **C** | PB1-F2 | NONE |
| | | |
| **A** | PA | I61M, T85A, T97N, D160E, Y176F, I201T, K262R, I322L, I354F, K391R, I505V |
| **B** | PA | V63M, D216N, K262R, S296T, Y305H, S388G, F520Y, Y535H, I543L, K615R |
| **C** | PA | V63M, D216N, S296T, Y305H, E327K, S388G, D479E, F520Y, Y535H, I543L, K615R |
| | | |
| **A** | PA-X | T97N, D160E, N245S |
| **B** | PA-X | V63M, K195R, K252R |
| **C** | PA-X | V63M, Q210P |
| | | |
| **A** | HA | V14A, N170D |
| **B** | HA | V5M, L6F, T10A, I11T, I67V, D104G, Q131R, N136D, S137Y, E142D, K156N, S157P, D170N, A172S, G179S, V190I, R205N, T211I, V226I, K228R, S336N, R339K, I390M, R420K, P505Q, I528V |
| **C** | HA | V5M, L6F, T10A, I11T, I67V, D104G, Q131R, N136D, E142D, K156N, S157P, D170N, A172S, G179S, V190I, R205N, T211I, V226I, K228R, R339K, I528V |
| | | |
| **A** | NP | D51E, M105V |
| **B** | NP | I41V, K48Q, V67A, A85P, V183I, I347L |
| **C** | NP | I41V, K48Q, A85P, V183I, I475V |
| | | |
| **A** | NA | K6R, T8I, I10T, Y44H, S70N, E77K, D259E, D287E, M289I, Q308H, V394I, S405T |
| **B** | NA | V8I, I13V, M29I, H36Q, I38F, G41R, N42F, T59A, S70P, L120M, I143V, K197N, R200G, E239K, I269M, K312T, K332R, H346N, T361N, G362N, E366D, D431E |
| **C** | NA | V8I, I13V, M29I, H36Q, I38F, G41R, N42L, T59A, S70P, L120M, I143V, K197N, R200G, V221I, E239K, I269M, K312T, K332R, H346N, T361N, G362N, E366D, D431E |
| | | |
| **A** | M1 | NONE |
| **B** | M1 | V115I |
| **C** | M1 | NONE |
| | | |
| **A** | M2 | NONE |
| **B** | M2 | W41C |
| **C** | M2 | NONE |
| | | |
| **A** | NS1 | S48I, S83P, L147I, D171N, G210E, V226I |

| | | |
|---|---|---|
| **B** | NS1 | S3P, G47S, I64V, S73P, A82T, A86T, R88H, R100K, R118K, N127T, F138L, D139N, L166F, I194V, R200K, S206C, N207D, D209G, P212T, P215S |
| **C** | NS1 | G47S, S73P, A82T, A86T, R88H, R100K, R118K, D125N, F138L, D139N, L166F, I194V, S206C, N207D, D209G, P212T, P215S |
| | | |
| **A** | NEP | G53R, G63E |
| **B** | NEP | S3P, K39R, A49V, M52V, D54E, E75D, N92S |
| **C** | NEP | K39R, M52V, D54E, E75D |
